# Advancing marine invertebrate cell line research: four key knowledge gaps

**DOI:** 10.1007/s11626-025-01029-y

**Published:** 2025-03-28

**Authors:** Baruch Rinkevich, Shirley A. Pomponi

**Affiliations:** 1 Israel Oceanographic & Limnological Research, National Institute of Oceanography, Tel Shikmona, 3108001 Haifa, Israel; 2 Florida Atlantic University, Harbor Branch Oceanographic Institute, Fort Pierce, FL USA; 3 Wageningen University, Bioprocess Engineering, Wageningen, The Netherlands

**Keywords:** Marine invertebrates, Cell cultures, Knowledge gap, Stemness, Omics, Media, Cell types

## Abstract

Although cell cultures from marine invertebrates have great potential as valuable tools in various scientific fields, nearly all attempts to culture these cells in vitro have consistently failed, and the reasons for this remain unclear. The ongoing failure to develop stable, long-term cell cultures from marine invertebrates, despite varied species and methods employed, highlights significant knowledge gaps in understanding their in vitro requirements. These gaps impede progress, underscoring the complexity of marine invertebrate cells and the need for innovative approaches to overcome challenges in the field. When reviewing recent literature on the key data deficiencies and challenges behind the failure to develop marine invertebrate cell cultures, we identified and discussed four major knowledge gaps: (1) optimizing culture media, (2) strategies to extend stemness of isolated cells, (3) using “omics” to enhance cell culture, and (4) selecting suitable cell types for in vitro cultures. Bridging these gaps is crucial for advancing marine invertebrate cell culture systems. Yet, given the current state-of-the-art, addressing these gaps and advancing the discipline necessitate comprehensive, integrated, and species- or cell-specific strategies, along with close collaboration among laboratories working on diverse species.

## Introduction

Marine invertebrate cells cultured in vitro hold immense potential as valuable tools across diverse scientific disciplines and applied fields, including biological/environmental studies (e.g., Rosner *et al*. 2021, 2024), economical applications, including cultivated seafood (e.g., Musgrove *et al*. 2024a; Rotter *et al*. 2024) and medical research (e.g., Datta *et al*. 2015). The uses of cell cultures from marine invertebrates further offer alternatives to animal experimentation, as well as opportunities for biotechnological innovation and physiologic/metabolic studies. Invertebrates, which comprise over 95% of all animal species, represent a significant resource for such research. Among them, the marine invertebrates that are known to occur in all habitats of the marine/oceanic domains, are estimated to account for 30–35% of all animal species (Ormond *et al*. 1997; Collier *et al*. 2016) and are noted for their dominance in marine ecosystems and their vast taxonomic diversity. Encompassing over 20 phyla and representing 92% of ocean species (Chen 2021), marine invertebrates provide a diverse range of cell and tissue types, which exhibit significant variation both between and within phyla. These cells further display remarkable morphogenetic potential, including multipotency, totipotency, and even neoplastic behaviors (Robert 2010; Rinkevich *et al*. 2022). This potential underpins their high in vivo plasticity, including dynamic changes in structures, cell proliferation, regeneration, and lineage differentiation, which can vary considerably even among closely related groups.

While mammals account for only 0.4% of existing multicellular taxa, they dominate cell culture research, with 97% of available cell lines originating from mammalian species (Bairoch 2018), highlighting a disproportionate scientific interest. In the same way, efforts to culture marine invertebrate cells in vitro are not a just a recent development, with research in this area dating back nearly six decades (Rannou 1968, 1971; Gomot 1971; Vago 1971). The literature reveals that research efforts have predominantly focused on six marine groups, the Porifera, Cnidaria, Crustacea, Mollusca, Echinodermata, and Urochordata (Vago 1971; Bayne 1998; Mothersill and Austin 2000; Potts *et al*. 2020; Domart-Coulon and Blanchoud 2022), with a focus on developing basic techniques for cell isolation and culture. With over 500 publications on aquatic invertebrate cell cultures (Domart-Coulon and Blanchoud 2022), almost all attempts to establish stable, long-term cell cultures from marine invertebrates have consistently failed, with the reasons remaining unclear (reviewed in Vago 1971; Bayne 1998; Rinkevich 1999, 2005, 2011; Mothersill and Austin 2000; de Caralt *et al*. 2007; Yoshino *et al*. 2013; Cai and Zhang 2014). With the exception of a recent breakthrough in developing a cell line from sponges (Hesp *et al*. 2023), no enduring or proliferative cell lines have been successfully established for aquatic invertebrates. While these studies contributed foundational insights, they frequently encountered significant challenges, such as sustaining cell viability and achieving long-term proliferation. This challenge is further amplified by the lack of published records on failed methodologies, as highlighted by Grasela *et al*. (2012), or opportunistic microorganisms being mistaken for invertebrate cells (Rinkevich 1999). The reluctance to publish unsuccessful experiments stems from their perceived unsuitability for most scientific journals that has hindered progress by limiting insights into the challenges and obstacles in marine invertebrate cell culture.

The general persistent lack of success in developing cell cultures from marine invertebrates, despite diverse species and experimental methodologies, underscores critical knowledge gaps in understanding the in vitro requirements and cellular mechanisms of these cells. These gaps further hinder the development of novel strategies to address persistent challenges in the field, emphasizing the complexity of marine invertebrate cellular systems and underscores the need for innovative approaches to overcome these barriers.

**Figure 1.**
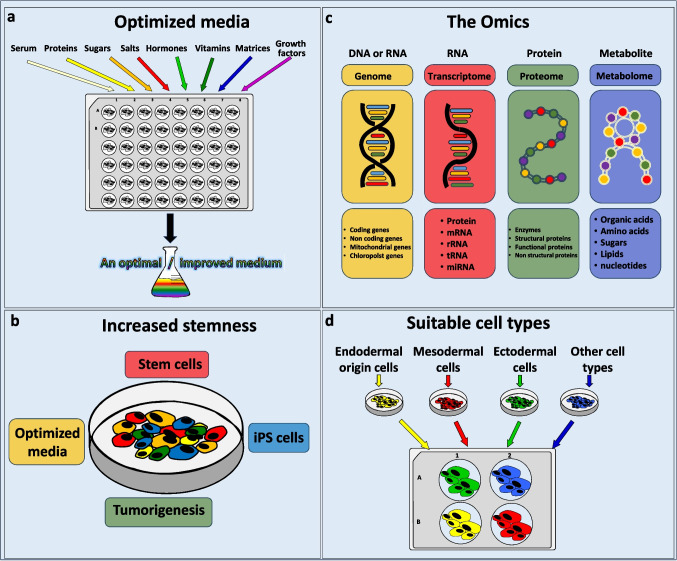
Four key knowledge gaps identified as barriers to the successful development of marine invertebrate cell cultures.

We have reviewed recent literature on prevalent data deficiencies and learning shortfalls underlying the failures in the development of cell cultures from marine invertebrates. This review highlights four major knowledge gaps (Fig. 1): (1) the need for specific and optimized improvements in culture media; (2) strategies to extend the limited in vitro stemness of isolated cells; (3) leveraging “omics” approaches for advancing cell culture techniques; and (4) establishing clear criteria for selecting the most suitable cell types for in vitro cultures. Addressing these gaps, as discussed below, is essential for advancing the field and unlocking the full potential of marine invertebrate cell culture systems.

## The need for specific and optimized improvements in culture media (Fig. 1)

Culture medium is the cornerstone of developing any cell line. Numerous attempts have been made to develop culture media for marine invertebrates, with the most common strategy involving the optimization of commercially available (or “off-the-shelf” (OTS)) mammalian or insect cell culture media (see Table 1). For mammalian cell culture, the demand for more consistent, chemically defined media led to the development of media based on nutritional biochemistry and body fluids analyses (Freshney 2000). Not unlike these first attempts to develop mammalian cell culture media, some researchers have tried to create de novo synthesis of media based on the extracellular milieu in vivo, such as hemolymph for mollusks or filtered sea water for ctenophores and cnidaria (Table 1). However, these efforts have not yielded better results than using optimized OTS media, with no significant improvement in cell survivability or proliferation (for a review, see Balakrishnan *et al*. 2022).

**Table 1.** Cell culture media, cell types, and culture applications for marine invertebrate cell culture since 2014. Note that all OTS media have been adjusted for osmolarity, and most media contained antibiotics

Organisms	Culture media	Cell types	Application	Reference
Porifera: numerous species of Demospongiae	M-199, M1, OpM1	Archaeocyte-enriched fractions in early studies; mixed cells in last decade	*In vitro* production of marine natural products	Munroe *et al*. (2019), Conkling *et al*. (2019), Hesp *et al*. (2023)
Ctenophora: *Mnemiopsis leidyi*	Filtered seawater, ctenophore mesoglea serum	Ectodermal cells, smooth muscle cells, digestive cells, sperm cells	Cellular differentiation and physiology; cellular basis of regeneration	Vandepas *et al*. (2017)
Cnidaria: *Anemonia viridis*	Grace’s modified insect medium	Endodermal and gastrodermal cells from regenerating tentacles, cnidocytes, dinoflagellate symbionts	Effect of thermal stress	Ventura *et al*. (2018)
Cnidaria: *Nematostella vectensis* and *Pocillopora damicornis*	Leibovitz’s L-15 medium	Cnidocytes, nematosomes, putative gastrodermal and epidermal cells	Model development: functional cell biology	Nowotny *et al*. (2021)
Cnidaria: 5 species of octocorals	Autoclaved filtered seawater	Aggregates of glandular cells, granulocytes, vacuolocytes, ciliated cells, several non-characterized cell types	Models to study biomineralization	Huete-Stauffer *et al*. (2015)
Crustacea: Indian mud crab *Scylla serrata*	Leibovitz’s L-15 medium, M 199, Grace’s insect medium, MEM, DMEM, TC-100 insect medium, IPL-41 insect medium, RPMI	Primary muscle cells, hemocytes	Effects of heavy metals; diseases	Sivakumar *et al*. (2019)
Mollusca: 23 species of bivalves, gastropods, cephalopods	Eagle’s MEM, Medium 199, Leibovitz’s L-15 medium	Stem-like cells in mantle; hemocytes; epithelial cells of gills and digestive gland; digestive gland cells; neoplastic cells	Primarily short-term experiments focused on specific applications	Reviewed by Balakrishnan *et al*. (2022)
Mollusca: *Crassostrea virginica*	Leibovitz’s L-15 medium or Opti-MEM	Derived from explants of heart, gill, mantle, adductor muscle, gonad, digestive gland	Identification of novel cell morphologies for studies of ecotoxicology, virology, immunology, disease	Potts* et al*. (2020)
Echinodermata: *Antedon mediterranea*	Leibovitz’s L-15 medium	Undifferentiated (amoebocytes, coelomocytes) and pluripotent cells (phagocytes, granulocytes, dedifferentiated cells)	Cell phenotypes responsible for arm regeneration	Di Benedetto *et al.* (2014)
Echinodermata: several species of holothurians	Leibovitz’s L-15 medium	Cells from regenerating gut rudiments: spherical-, oval-, spindle-shaped cells	Developmental and regenerative biology	Bello *et al*. (2015)
Echinodermata: *Apostichopus japonicus*	Leibovitz’s L-15 medium	Intestinal cells: round and spindle-shaped		Wang *et al*. (2020)
Tunicata: *Botryllus schlosseri*	5 variations of “tunicate growth medium” (TGM): ASW, DMEM/F-12, RPMI 1640	blood cells (several types)	*In vitro* production of biopharmaceuticals; stem cells; developmental biology	Qarri *et a*l. (2023)

The selection of media for marine invertebrate cell culture is often based on what has been used by other researchers trying to culture cells from the same species, rather than on the specific nutritional requirements of the organisms or cell types. For example, there is still limited understanding of the in vitro nutritional requirements for proliferation and maintenance of sponge cells (Cai and Zhang 2014). Conversely, for many molluscan species, the knowledge of osmolality, growth factors, and other requirements is available and could be applied to the development of cell lines (reviewed in Balakrishnan *et al*. (2022)).

Mammalian and insect cell culture media were optimized to meet the specific nutritional needs of the cell type being cultured. They range from simple composition (e.g., Eagle’s minimum essential medium (EMEM)) to more complex options (e.g., Medium 199 (M199), Dulbecco’s minimum essential medium (DMEM), RPMI 1640, Ham’s (DMEM/F12), and Grace’s insect medium). Leibovitz’s L-15 medium, for example, was designed with a different buffering system to support cells in a CO_2_-free atmosphere. While most OTS culture media selected for marine invertebrate cell cultures tend to be more complex, with some even combining two OTS media (Qarri *et al*. 2023), it is likely that the rationale behind their initial development and the specific cell types they were designed to support may not have been fully considered during the selection process (see Yao and Asayama 2017, for a detailed review of cell culture medium development).

Two other important variables to consider in developing culture media are the use of serum and antibiotics. While contamination of primary cultures, primarily noted in the early days following isolation (Rinkevich 1999; Grasela *et al*. 2012; Pers. Obser.), will not be addressed here, it is evident that primary cultures are prone to a wide range of contaminants, including prokaryotic and eukaryotic organisms (Grasela *et al*. 2012). Serum, such as calf, fetal bovine, and horse serum, is often used in cell cultures because it provides essential growth factors, proteins, lipids, hormones, and other compounds that can stimulate cell division. However, the downside of using a serum is that the composition is undefined, leading to variability between different batches.

Antibiotics are commonly employed to control contaminants, with most researchers using some combinations of penicillin, streptomycin, gentamicin, and/or amphotericin B to target bacteria and fungi. However, these antibiotics are ineffective against unicellular eukaryotic parasites, such as one of the most persistent contaminants, thraustochytrids (Rabinowitz *et al*. 2006; Qarri *et al*. 2021), and do not address other potential contaminants, such as viruses and mycoplasma. One notable exception is Nowotny *et al*. (2021), who added plasmocin as a prophylactic measure in cnidarian cell cultures (Table 1). Just as culture media are optimized by testing various combinations of components and assessing cultures for viability and proliferation, a similar approach should be applied to determine the most effective antibiotic (or combination thereof) to control the specific type(s) of contamination while preserving the viability and proliferative capacity of the cultured cells (e.g., Grasela *et al*. 2012). For applications such as the in vitro production of marine-derived pharmaceuticals, it will eventually be necessary to develop antibiotic-free cell lines. The importance of maintaining strict aseptic technique, particularly during cell isolation, along with regular media changes, cannot be overemphasized (Grasela *et al*. 2012).

Table 1 lists some media used over the last decade for cell culture of sponges, ctenophores, cnidaria, crustaceans, mollusks, echinoderms, and tunicates. It also highlights the cell types isolated for culture, and the applications explored in these studies. The most common medium selected over the past decade is Leibovitz’s L-15 medium, likely due to its design for incubating cells in a CO_2_-free atmosphere. However, recent success in culturing sponge cells in variations of Medium 199 (Conkling *et al*. 2019; Hesp *et al*. 2023) suggests that, at least for sponges, a medium specifically designed for a CO_2_-free atmosphere may not be required. It is worth noting that cells from species with photosynthetic symbionts, such as some cnidaria, can be co-cultured with their symbionts and maintained without the use of other nutrients in the culture medium (Huete-Stauffer *et al*. 2015). The below review of culture medium knowledge gaps focuses on two groups, sponges and tunicates, for which different approaches to medium development have been explored, yielding some of the most promising results.

### Sponges

Cell culture medium is a complex mixture of nutrients and growth factors in which the contribution of each component is crucial for the long-term maintenance and growth of each specific cell type (Price 2017). To optimize culture media for sponge cells, researchers have utilized various methods, including the Plackett–Burman design with response surface methodology and uniform design (Zhao *et al*. 2005), one-dimensional search approaches (Willoughby and Pomponi 2000), and genetic algorithms (Munroe *et al*. 2019). These studies led to improvements in metabolic and esterase activity, DNA and protein content, and, in some cases, increases in cell numbers. Yet, the developed media are generally species-specific, as the optimized medium formulations did not yield the same results when applied to different sponge species (Zhao* et al*. 2005; Conkling *et al*. 2019). This species-specificity remains a common challenge in sponge cell culture.

Designing and optimizing culture media require methods that can handle numerous components with complex interactions. Genetic algorithms (GAs) have been used to optimize parameters like insect cell growth (Marteijn *et al*. 2003), microalgal cells (Camacho-Rodríguez *et al*. 2015; López-Rosales *et al*. 2015), sponge cell metabolism (Munroe *et al*. 2019), and yeast production (Etschmann *et al*.^ 2004^). GAs optimize multiple parameters (medium components) without needing detailed information on compound concentrations or cellular metabolism (Marteijn *et al*. 2003). They simulate natural selection by randomly generating a “population” of experimental conditions, allowing the best performers to “mate” and evolve through crossover and mutations until optimal results are achieved (Ranganath *et al*. 1999; Weuster-Botz 2000; Marteijn *et al*. 2003).

The GA offers several advantages over other medium optimization methods for sponge cell culture. It allows for the selection of component concentration from a range of values, without needing prior knowledge of interaction or cytotoxicity, enabling the evaluation of more components in less time (Marteijn *et al.* 2003; Etschmann *et al.* 2004). Typically, a GA can reach near-optimum results in under 10 generations (Weuster-Botz 2000), testing hundreds or even thousands of medium compositions. For example, optimizing the amino acid composition for *Dysidea etheria* sponge cells took just four generations, leading to the creation of the first optimized variation of Medium 199, M1 (Munroe *et al*. 2019). Using the same GA with M1 medium as the base resulted in a new medium variation, OpM1, with optimized concentrations of vitamins, trace elements, lipids, growth factors, and serum (Hesp *et al*. 2023). Both M1 and OpM1 improved metabolic activity and cell proliferation in primary cell cultures compared to earlier media. Suprisingly, although M1 did not result in cell proliferation in the species for which it was developed, a test of the medium on 12 randomly selected species from diverse orders resulted in significant cell proliferation of nine species (Conkling *et al*. 2019). When cells of the deep-water sponge *Geodia barretti* were cultured with OpM1, they exhibited a rapid growth rate and nearly 100 population doublings (Hesp *et al*. 2023). Ironically, the two species used in the Pomponi lab for decades (*Dysidea etheria*, *Axinella corrugata*) did not proliferate in either of these media, emphasizing the importance of not limiting medium optimization to the researcher’s “model species.”

### Tunicates

At the other end of the phylogenetic spectrum are the tunicates, with the species *Botryllus schlosseri* being a longstanding focus of marine invertebrate cell culture research for decades. Studies on this species have explored the initiation of primary cell cultures from embryos, epithelial cells, and circulating blood cells, assessing the effects of growth factors on primary cultures, establishing a defined medium for blood cells, and evaluating stemness signatures in epithelial monolayers (Rinkevich and Rabinowitz 1993, 1994, 1997, 2000; Rabinowitz and Rinkevich 2004, 2005, 2011; Rabinowitz *et al*. 2009; Qarri *et al*. 2023). Despite the occurrence of several types of stem cells in *B. schlosseri* blood, epithelium, and embryos (Rinkevich and Rabinowitz 1993; Rabinowitz and Rinkevich 2004, 2011; Rabinowitz *et al*. 2009; Qarri *et al*. 2022), no cell lines have been established.

Qarri *et al*. (2023) recently conducted a comprehensive analysis of *B. schlosseri* blood cell proliferation in five different medium formulations, with “tunicate growth medium” (TGM) as the base. This TGM base contains L-glutamine, HEPES buffer, sodium pyruvate, and fetal bovine serum. Five variations of the TGM base were prepared with or without OTS media (DMEM/F-12 (Ham) or RPMI 1640), with or without artificial seawater (ASW), and each with different combinations of antibiotics, as follows: TGM1-DMEM/F-12 (Ham), penicillin, streptomycin; TGM2-RPMI 1640, penicillin, streptomycin; TGM3-ASW, penicillin, streptomycin, amphotericin B, gentamicin; TGM4-DMEM/F-12 (Ham), ASW, gentamicin, penicillin, streptomycin; and TGM5-DMEM/F-12 (Ham), ASW, penicillin, streptomycin (Qarri *et al*. 2023). Cultures were monitored using confocal microscopy, with each cell type distinguished by its autofluorescence emission intensity across the blue, green, red, and far-red channels. Proliferation was measured through immunofluorescence detection of proliferating cell nuclear antigen (PCNA^+^). Within the first week, an increase in cell proliferation was observed among distinct blood cell types. The distribution of cell types varied across the five media and changed over time. PCNA^+^ activity also varied among the media and changed over time, with various blood cell types proliferating at different times. Within 1 month, medium-specific primary cultures were developed. This is an important finding, providing compelling evidence for the potential to develop cell-type specific cultures for tunicates and other marine invertebrates.

While the development of culture media typically prioritizes cell proliferation (expansion), it may be necessary to culture differentiated cells. This would require switching from a medium that supports expansion to one that promotes differentiation (for review, see Yadav *et al*. 2020). The results of Qarri *et al*. (2023) provide a promising example of the potential to use different media to culture various marine invertebrate cell types, each with distinct proliferative capacities and functions.

## Strategies to extend the limited *in vitro* stemness of isolated cells (Fig. 1)

Stemness refers to the characteristics and properties that define stem cells, particularly in the context of their ability to self-renew. Preserving the “stemness” of stem cells, and remaining undifferentiated, is essential for successful application in a wide range of scientific and medical fields (Hurtley 2015), as in the cell culture field. In mammalian systems, the in vitro culture of stem cells has progressed rapidly, driven by innovations like niche-based methods (Pal and Das 2017), 3D spheroid and organoid cultures (Yen *et al*. 2023), bioreactors, 3D scaffolds (Yi *et al*. 2018), and coating culture dishes with attachment-inducing components (e.g., gelatin, Matrigel, collagen; McKee and Chaudhry 2017). These methods address many limitations of traditional 2D cultures, offering significant potential to enhance stem cell therapeutic applications in regenerative medicine and beyond.

The application of stemness technologies to marine invertebrate cell cultures is still in its early stages, revealing a significant knowledge gap. Unlike the significant advancements in vertebrate cell cultures and cell lines, decades of research have yet to produce a permanent cell line from marine invertebrates, with only one recent successful approach involving sponge cells (Hesp *et al*. 2023). Under in vitro conditions, isolated cells from many marine invertebrate taxa cease division and enter quiescence within 24–72 h. To address this research challenge, it is essential to modify the universal quiescent state, enabling certain cells to acquire pluripotency, allowing for indefinite division and achieving immortality (Rinkevich 1999, 2005, 2011; Anoop *et al*. 2021; Sudarshan *et al*. 2023). Potential stem cell activities can further be elucidated using assays that involve regeneration processes in marine invertebrates (Rinkevich *et al*. 2010; Levanoni *et al*. 2024; Musgrove *et al*. 2024b). Harnessing stem cells from marine invertebrates for extended in vitro stemness offers a promising approach to overcoming current obstacles, though it remains a challenging field. Several authors (de Caralt *et al*. 2007; Sun *et al*. 2007; Odintsova 2009; Rinkevich 2011; Domart-Coulon and Blanchoud 2022; Mohajer *et al*. 2024) have suggested that extended in vitro stemness could be achieved using (a) adult stem cells from marine organisms or (b) methodologies developed for mammalian-induced pluripotent stem (iPS) cells or for tumorigenesis (e.g., Anoop *et al*. 2021; Sudarshan *et al*. 2023; not discussed here).

Marine invertebrates, including sponges, cnidarians, flatworms, crustaceans, mollusks, echinoderms, and ascidians, possess substantial pools of adult stem cells that are vital for maintenance, regeneration, and asexual reproduction (Ballarin *et al*. 2018, 2022; Rinkevich *et al*. 2022). Yet marine invertebrate stem cells (MISC) exhibit a wide range of occurrences and phylum-specific characteristic morphologies and behaviors, with the typical well-characterized sponge archaeocytes and choanocytes, hydrozoan I-cells, platyhelminth and acoel neoblasts, and tunicate hemoblasts (Domart-Coulon and Blanchoud 2022; Ereskovsky *et al*. 2022, 2024; Rinkevich *et al*. 2022). Representing up to 40% of an organism’s cells, they play crucial roles in processes such as whole-body regeneration, dormancy, agametic asexual reproduction, and indeterminate growth, further recognized as valid units of selection (Rinkevich *et al*. 2009, 2022; Vanni *et al*. 2022b). MISC arise at various life stages, displaying both differentiated and undifferentiated phenotypes and often exhibiting amoeboid movement (Domart-Coulon and Blanchoud 2022; Rinkevich *et al*. 2022). Typically, pluri- or totipotent, MISC may express germ-cell markers, but they usually lack germ-line sequestration and do not reside in distinct niches (Domart-Coulon and Blanchoud 2022; Martinez *et al*. 2022; Rinkevich *et al*. 2022; Vanni *et al*. 2022b).

As of the above, the identification, isolation, and characterization of stem cells in aquatic invertebrates remain major technical challenges, often requiring species-specific approaches and the use of validated stem cell markers (Domart-Coulon and Blanchoud 2022; Rinkevich *et al*. 2022). For instance, in the branching coral *Stylophora pistillata*, stem cells were not identified in the cell atlas derived from both larval and adult tissues, despite using enzymatic or mechanical dissociation methods and known markers (Levy *et al*. 2021). Furthermore, research efforts have established protocols for isolating identified stem-like cells in only a few species (e.g., Hayashi *et al*. 2006; Sun *et al*. 2007; Hemmrich *et al*. 2012; Reyes-Bermudez *et al*. 2021). Another promising approach involves genetically modified marine invertebrates, where transgenic reporters for stemness properties can be engineered (e.g., in *Hydra*, Juliano *et al*. 2014). However, tapping into the diverse range of specific techniques developed for other taxa (including different species within the same phylum) holds great potential for broader generalization and application.

Few publications to date have specifically focused on developing cell cultures from marine invertebrates using purified or enriched stem cells (e.g., Zhang *et al*. 2003; Sun *et al*. 2007; Reyes-Bermudez *et al*. 2021). Mohajer *et al*. (2024) recently compiled diverse insights into MISC detection by reviewing studies that focus on identifying adult stem cells in various marine invertebrate organisms, without citing papers that applied MISC in cell cultures. Yet, in sets of 4-day primary sponge cell cultures, purified archaeocytes (adult stem cells) from *Hymeniacidon perleve* showed a significant 2.5-fold increase in total cell number, showcasing their potential for developing sponge cell cultures to produce valuable sponge-derived pharmaceuticals (Sun *et al*. 2007). Following the same rationale, Zhang *et al*. (2003) utilized sponge primmorph cultures derived from an archaeocyte-dominant cell population that were enriched via a Ficoll gradient, rather than the typical mixed-cell population method. This approach resulted in significant increases in DNA synthesis, cell proliferation (up to threefold), cell growth (up to fourfold), and, in long-term cultures, enhanced metabolic activity of the primmorphs. Reyes-Bermudez *et al*. (2021) presented gene expression profiles of cultured coral (*Acropora digitifera*) cells, highlighting the regulatory gene networks involved in pluripotency and differentiation. In cultures derived from the coral’s tip fragments (the colony’s fastest-growing tissues), the in vitro transcription profile resembled that of early larvae, with overexpression of orthologs to premetazoan and *Hydra* stem cell markers, along with transcripts associated with cell division, migration, and differentiation.

iPS cells are a type of stem cells generated by reprogramming somatic (adult) cells to a pluripotent state. The most commonly used genes for reprogramming somatic cells into iPS cells (known as the Yamanaka factors) are Oct4, Sox2, Klf4, and c-Myc, all found in marine invertebrates (Rinkevich *et al*. 2022; Vanni *et al*. 2022a). Yet, there is a greater knowledge gap regarding the use of iPS cells for marine invertebrate cell cultures. In vertebrate systems, the generation of iPS cells from somatic cells mimics ontogenetic processes, but may not parallel natural in vivo mechanisms (Chatterjee *et al*. 2015). Yet, the iPS technology, where somatic cells have been modified to acquire an embryonic stem-cell-like capacity, holds great promise for developing marine invertebrate cell cultures, as it can overcome the major challenge of cellular quiescence observed in vitro after isolating adult cells (Rinkevich 2011). With this in mind, more research is required to evaluate the potential of established mammalian techniques in the marine invertebrate cells arena, trying to employ endogenous homologs of Yamanaka factors. Clearly, more efforts into marine invertebrate pluripotency may add to this subject as additional Yamanaka-like factors could be uncovered. Research on iPS cell culture conditions in the mammalian systems has primarily focused on two key areas: enhancing reprogramming efficiency and quality, and developing human iPS cell culture systems for clinical applications (Mochiduki and Okita 2012). Following the above, iPS cells derived from marine invertebrates could then remain in a primitive state and readily proliferate into various cell lineages, leveraging their innate capacity for self-renewal and differentiation to facilitate the creation of immortal cell lines. To date, no studies have applied iPS technology to develop cell cultures from marine invertebrates. In addition to the use of stem cells and the iPS cells methodology for extending the limited in vitro stemness, recent publications (Anoop *et al*. 2021; Sudarshan *et al*. 2023) further attempted the approaches of cell hybridization and ectopic expression of mutated genes.

## Leveraging “omics” approaches for advancing cell culture technique (Fig. 1)

In the last two decades, rapid advancements in mammalian cell culture and cell engineering have highlighted the importance of using “omics” techniques to better understand cellular mechanisms and pathways, including apoptosis, cell proliferation, cell stimulation or quenching, and the effects of physicochemical environments (Rinkevich 2005; Kuystermans *et al*. 2007; Čuperlović-Culf *et al*. 2010; Zhang *et al*. 2013; Farrell *et al*. 2014; Liu *et al*. 2019; O’Brien and Hu 2020), as well as for applied approaches such as cancer research (Berg *et al*. 2017) and drug development (Buriani *et al*. 2012). The “omics” techniques that are integrated with cell culture development enable the detection of comprehensive changes in expression at the transcriptomic (mRNA), proteomic (protein), and metabolomic (metabolic) levels, among others. These usages improve understanding cell culture systems, as well as the statuses (in expression levels) depicted between different laboratories worldwide on the same cell line or among successive passages in an established cell line, such as HeLa cells (Liu *et al*. 2019). The application of multi-omics techniques further improves the progression in cell culture optimization and bioprocess designs (Farrell *et al*. 2014). As an example, proteomics can reveal the overall extent of peptide expressions within a specific cell culture condition. When combined with transcriptomic and metabolomic approaches, proteomics provides valuable insights into how cells under in vitro conditions respond to the various repertoire of conditions. Metabolomics, which represents the global quantitative assessment of metabolites under in vitro conditions, provides crucial data for system-level analysis and modeling of biological processes when conducted alongside other “omics” measurements (Čuperlović-Culf *et al*. 2010). With advancements in current and emerging technologies, “omics” research may thus evolve to address more complex systemic questions and serve as a valuable tool in the development of cell cultures from marine invertebrates.

We identify a significant knowledge gap in the application of “omics” approaches for developing cell cultures from marine invertebrates. Recent advancements in next-generation sequencing techniques, particularly in single-cell transcriptomics, are allowing researchers to characterize stem-like cells across a growing number of taxa (e.g., Musser *et al*. 2021; Rinkevich *et al*. 2022), marking an essential first step toward their isolation and in vitro culture. Further, insights into in vivo tissue homeostasis, cell proliferation dynamics, and somatic stem cell niches (Martinez *et al*. 2022) can aid in selecting specific tissue spots with high proliferative potential. Yet, despite declining costs of sequencing and “omics” technologies and increasing efforts to characterize differentiated and stem cells in marine invertebrates, suggesting “omics” data will soon be available for most taxa (Domart-Coulon and Blanchoud 2022), there remains a shortage of studies demonstrating the broad accessibility of such data across nearly all marine invertebrate taxa. Further, while there is a broad range of “omics” studies focusing on marine invertebrates at the whole-organism level (e.g., Imbs *et al*. 2021; Romano *et al*. 2022; Kültz *et al*. 2024), very little has been directed towards the use of omics methodologies for the development of primary cell cultures from marine invertebrates (but see Kawamura *et al*. 2021), with limited scientific approaches.

One such approach is the work of Tsuchiya *et al*. (2023) that conducted transcriptomic analysis of primary lymphoid cells in kuruma shrimp (*Marsupenaeus japonicus*) to understand gene expression changes under their culture conditions. RNA sequencing at four culture time points (days 1, 3, 4, 6) identified three gene expression patterns: (1) downregulated on days 3–6, (2) upregulated on days 3–4, and (3) upregulated on day 6. Notably, the shrimp VEGF3 and its receptor showed significant downregulation, confirmed by qPCR. These findings highlighted the need for timely VEGF signaling supplementation into cultured cell medium to maintain stable, long-term shrimp lymphoid cell cultures.

Promising intermediate approaches that may open the road for cell culture studies involve the indirect use of “omics” techniques for in vitro studies of marine invertebrates. This is particularly valuable for developing in vitro ecotoxicology models, such as assessing at the transcriptomic level the effects of heavy metals on bivalve hemocytes (de Boissel *et al*. 2017) or at the proteomics level, the toxicity of nanoparticles on bivalve tissues (De Felice and Parolini 2020). The same implies to approaches searching for the integration between immune cells and stem cells in marine invertebrates (Ballarin *et al*. 2021) or elucidating proteomic outcomes from purified cell types of crayfish hemopoietic tissue (Söderhäll and Junkunlo 2019). The application of “omics” technologies to marine invertebrate cell cultures is still in its early stages, highlighting a significant knowledge gap.

## Establishing clear criteria for selecting the most suitable cell types for *in vitro* cultures (Fig. 1)

As noted earlier, the selection of species plays a crucial role in the success of establishing a cell line, particularly considering the species-specificity of the culture media developed for marine invertebrate cell culture (Zhao et al. 2005; Conkling *et al*. 2019). If the goal of the research is to develop primary cultures or cell lines for a specific application (rather than developing a cell line per se), the choice of species (and cell types) could potentially impede the successful establishment of long-term cultures. A continued lack of success in cell proliferation using the researcher’s “model species” may warrant investigation of cells from other related species (Conkling *et al*. 2019).

Selection of cell types for marine invertebrate in vitro cultures may be based on the putative stemness of the cells, the intended use of the cultures, and/or cell lineage (Fig. 1). “Other cell types” (Fig. 1) includes cells for which their embryonic origin is not known and for which their morphologies change in cultures. For example, sponges do not have true germ layers (Ereskovsky and Dondua 2006) and sponge cells are able to transdifferentiate (Adamska 2018), which makes characterization of cell lineage challenging. Indeed, cell lines may not be necessary for short-term studies that can address hypotheses using primary cell cultures, and the optimization of culture variables will depend on the hypotheses being tested. For example, Andrade *et al*. (1999) used archaeocyte-enriched fractions of cells from the sponge *Teichaxinella morchella* (= *Axinella corrugata*), cultured for 48 h in Medium 199, to identify the biosynthetic origin of stevensine, a sponge alkaloid, using radiolabeled amino acid precursors. Balakrishnan *et al*. (2022) provide a comprehensive review of applied research in toxicology, pathology, and neurophysiology using primary molluscan cell cultures.

A critical factor to consider is the source of the cells (embryos, larvae, juveniles, or adults) which will depend on the type of cells required to develop a cell line or address a specific hypothesis. For example, if the goal is to isolate stem cells, embryos or larvae are the obvious choice. However, obtaining embryos or larvae from some species may be challenging if their reproductive cycles are unknown or they do not release larvae when taken from their natural environment.

The development of marine invertebrate cell cultures has traditionally focused on using tissues, organs, or part of the entire organism as the source of cells. For sponges, as an example, fragments of the whole animal are dissociated into single cells, some of which may revert into a stem-like state and/or transdifferentiate (Adamska 2018). In the cnidarians, however, when fragments of the entire specimen are dissociated into single cells, these cells remain in suspensions without a visible re-differentiation, usually staying in terminally differentiated state (Domart-Coulon and Blanchoud 2022). In contrast, when the ectodermal tissue is specifically isolated, then ectodermal monolayers are specifically developed (Rabinowitz *et al*. 2016). Table 1 provides a list of the most commonly used marine invertebrate cell types over the past decade for cell cultures development. Methods for isolation and separation are not the focus of this review; yet, they are described in the papers cited in Table 1.

There may be valuable insights to gain from recent efforts to develop cultivated meats in general, and cultivated crustacean meat, in particular (reviewed by Musgrove *et al*. 2024a). Since no crustacean cell lines currently exist, the focus has been on isolating and culturing adult muscle stem cells from lobster tail muscles, with some success (Jang *et al*. 2022). However, since this approach involves sacrificing the source organism, alternative methods are being explored to establish an immortalized cell lines with both proliferative and myogenic potential from non-lethal sources (Musgrove *et al*. 2024a). Among the most promising sources are hematopoietic stem cells, which are found in many marine invertebrates (Rinkevich *et al*. 2022) and that in crustaceans show some potential for differentiating into muscle cells (Musgrove *et al*. 2024a).

This leads back to a discussion of stemness and “omics.” To break through the bottleneck that has hindered the development of marine invertebrate cell lines may require more research to isolate and identify putative stem cells of the targeted species, the use of immunofluorescent antibodies to characterize both undifferentiated and differentiated cells to determine their proliferative capacity (Jang *et al*. 2022; Qarri *et al*. 2023; Musgrove *et al*. 2024a), and the use of “omics” technologies to refine the selection. Continuing to use the same trial-and-error approach without a better understanding of the proliferative and functional capabilities of the cells we are attempting to culture will only lead to incremental improvements. A breakthrough may come with the use of more sophisticated tools.

## Discussion

It is well documented that isolated cells from a wide range of marine invertebrates cease division in vitro within 24–72 h after isolation, entering states of cellular quiescence (Rinkevich 2011). This happens despite the fact that many marine invertebrates across a broad range of taxa (from sponges, cnidarians, and flatworms to urochordates) possess pluripotent and even totipotent adult stem cells throughout their lives. These cells are utilized not only for maintaining the animal’s body but also for biological processes like asexual reproduction, budding, and whole-body regeneration (Rinkevich *et al*. 2022). This widespread quiescent state must be altered so that at least some of the quiescent cells regain pluripotency, enabling them to divide and achieve immortality.

In the present overview, we identified and examined four key knowledge gaps in the development of cell cultures from marine invertebrates, including the optimization of culture media, the development of strategies to prolong the stemness of isolated cells, leveraging “omics” technologies to improve cell culture, and the selection of appropriate cell types for in vitro cultivation (Fig. 1). Given the current state-of-the-art in this field, addressing these gaps and perhaps even integrating approaches to address the gaps are critical for advancing marine invertebrate cell culture systems.

The literature reveals that the biological reasons for the failure to establish long-lasting and proliferating cell cultures from marine invertebrates remain unclear, highlighting the need for deeper insights. These insights may involve developing specialized approaches rather than relying on universal techniques, such as designing tailored culture media, employing specific cell dissociation protocols, selecting different cells, tissues, organs, or organisms for the study, and utilizing tissue fragments or adherent/non-adherent cells. The literature further depicts that it is preferable to customize these strategies for a particular model tissue, organ, or species, rather than adopting a one-size-fits-all approach (Vago 1971; Rannou 1971; Bayne 1998; Rinkevich 1999, 2005; Mothersill and Austin 2000; Conkling *et al*. 2019;  Potts *et al*. 2020; Balakrishnan *et al*. 2022; Domart-Coulon and Blanchoud 2022). This contrasts with the assumption that all cells across different taxa within the kingdom Animalia share similar nutrient requirements, are regulated by identical developmental and physiological-biochemical pathways, and are influenced by the same gene expression cascades (Pires-daSilva and Sommer 2003).

For decades, researchers have attempted to establish continuous cell cultures from marine invertebrates, with limited success (Bayne 1998; Rinkevich 1999, 2005; Mothersill and Austin 2000; Balakrishnan *et al*. 2022; Domart-Coulon and Blanchoud 2022). One obstacle to successful cell culture from these species may stem from the lack of published information on unsuccessful methodologies, leading to wasted time and effort on repeatedly reinventing similar methods and procedures (Grasela *et al*. 2012). Four key obstacles are outlined here, that include (1) optimizing culture media, (2) strategies to extend stemness of isolated cells, (3) using “omics” to enhance cell culture, and (4) selecting suitable cell types for in vitro cultures. Advancing the field and overcoming these and other challenges requires adopting holistic, targeted research approaches tailored to specific species or cell types, as well as fostering strong collaboration among laboratories studying diverse organisms.
